# The Road to Improved Assisted Living Quality: State Efforts and New Metrics

**DOI:** 10.1093/geroni/igaa057.2575

**Published:** 2020-12-16

**Authors:** Tetyana Shippee, Lindsay Schwartz

## Abstract

Assisted living (AL), a senior housing option that combines housing, support services, and health care, is recognized as one of the fastest-growing components of the long term care industry. AL is also a relatively expensive service, whether it’s paid for privately or publicly. Also, an increasing proportion of AL residents have diagnoses of Alzheimer’s and related dementias. However, little is known about AL quality, in part due to lack of measures. Quality of AL matters to residents, their families, and policy makers because AL is not only about the experience of receiving specific services, but about a place that many will call home. Concerns have been surfacing regarding the quality of AL, including poor staffing, inadequate teamwork, and poor management, which can negatively impact resident well-being and result in abuse and neglect in some cases. This symposium will feature four presentations that will review efforts from two states that have been actively developing measures to address AL quality: Oregon and Minnesota. We focus on new legislation in both states to address AL quality, the new metrics being adopted, and preliminary results. Individual presentations will describe 1) Oregon’s new quality measures for AL ;2) Oregon’s use of Resident VIEW project, including measures of person-centered care from the perspectives of residents; 3) Minnesota’s development of AL report card, and 4) development and results from MN pilot surveys of resident quality of life and family satisfaction in all licensed ALs in the state. Policy implications for other states and researchers will be discussed.

